# Genetically Thermo-Stabilised, Immunogenic Poliovirus Empty Capsids; a Strategy for Non-replicating Vaccines

**DOI:** 10.1371/journal.ppat.1006117

**Published:** 2017-01-19

**Authors:** Helen Fox, Sarah Knowlson, Philip D. Minor, Andrew J. Macadam

**Affiliations:** Division of Virology, National Institute for Biological Standards and Control, Potters Bar, Hertfordshire, United Kingdom; University of California, Irvine, UNITED STATES

## Abstract

While wild type polio has been nearly eradicated there will be a need to continue immunisation programmes for some time because of the possibility of re-emergence and the existence of long term excreters of poliovirus. All vaccines in current use depend on growth of virus and most of the non-replicating (inactivated) vaccines involve wild type viruses known to cause poliomyelitis. The attenuated vaccine strains involved in the eradication programme have been used to develop new inactivated vaccines as production is thought safer. However it is known that the Sabin vaccine strains are genetically unstable and can revert to a virulent transmissible form. A possible solution to the need for virus growth would be to generate empty viral capsids by recombinant technology, but hitherto such particles are so unstable as to be unusable. We report here the genetic manipulation of the virus to generate stable empty capsids for all three serotypes. The particles are shown to be extremely stable and to generate high levels of protective antibodies in animal models.

## Author Summary

There is a need for safe production of polio vaccines as eradication is approached. Empty capsids in a native conformation are produced by poliovirus and other picornaviruses seemingly as a necessary part of the assembly process, possibly to provide a reservoir of subunits in a form that is resistant to cellular pathways that target unfolded or hydrophobic motifs for proteolytic degradation. Normally they are not very stable prior to genome encapsidation but more stable forms, if they existed, could potentially be useful as vaccines. Genetic variants that increase empty capsid stability have been identified and by artificially combining several in one sequence the evolutionary constraints have been bypassed, with the resulting stable empty capsids representing essentially dead-end products. They induce antibody efficiently and are stable on storage. Empty capsids can be produced by recombinant expression which, if it were efficient enough, could provide a source of immunogenic particles suitable for use as vaccines without the need for live virus at any stage of production. This would be ideal for a post-eradication world.

## Introduction

The Global Polio Eradication Initiative (GPEI) is the largest intervention against a single disease in history. Naturally occurring wild type 2 poliovirus has not been seen globally since 1999, type 3 since 2012 and Afghanistan and Pakistan are the only countries where endemic type 1 virus is still circulating [1]. The situation is complex however [2]; there are sporadic importations into countries where endogenous circulation had been previously interrupted and the use of the live vaccine is problematic as the strains can revert to virulent transmissible forms (circulating vaccine derived polio viruses, cVDPV) or be excreted over long periods from immune deficient patients exposed to the live attenuated vaccine (immune deficient vaccine derived polioviruses, iVDPVs) [3]. Processes are being put in place to ensure that when polio is eradicated it does not re-emerge and these encompass the containment of work on the live virus and of production of the vaccine needed to ensure that coverage is maintained to guard against possible re-emergence [4]. The issues have been brought into focus by the WHO decision to withdraw the type 2 component from Oral Polio Vaccine (OPV) from mid-2016, and to introduce at least one immunisation with Inactivated Polio Vaccine (IPV). This means that work on all type 2 viruses must be contained at a higher level [4, 5]. This will include IPV production where colossal amounts of virus are grown and subsequently inactivated with formalin; the strains in current use are mainly wild types known to be able to cause poliomyelitis in humans, although two companies have licensed products based on the live attenuated Sabin strains used in OPV. Safe production of IPV is essential and one way to do this is to devise viable production strains that are intrinsically safer [6, 7, 8]. An alternative described here involves production of empty viral particles with the correct antigenic and immunogenic properties which could be expressed by recombinant technology and not involve infectious virus at any stage. The main difficulty with such an approach is that naturally occurring empty particles are extremely unstable. We have therefore engineered empty viral particles based on the three existing IPV production strains to give virus-like particles (VLPs) that are at least as stable as the inactivated virus in IPV, have the same antigenic structures as the native viruses and are at least as immunogenic as IPV. Provided they can be produced at the appropriate scale this platform has major advantages compared to the current procedures in terms of both safety and the properties of the product.

## Results

### Identification of stabilising mutations

#### (i) Type 3 Sabin vaccine strain

One of the two main attenuating mutations in the type 3 Sabin vaccine strain is in the structural protein VP3 at residue 91, which is a serine in the wild type Leon strain and a phenylalanine in the vaccine strain [2]. Amino acid 91 of VP3 lies at the interface between protomers [9] and makes virus growth and capsid assembly temperature sensitive (*ts*) in vitro [10]. Infants given oral polio vaccine eventually excrete virus in which the mutation is either reverted or suppressed and the suppressor mutations have been shown to increase the stability of capsid assembly intermediates [2]. Over the years we have identified additional mutations [11] that suppress the *ts* phenotype by growing the type 3 vaccine strain in HEp2C cells at semi-permissive temperatures. Eight of the mutations identified that may also increase capsid stability are given in Table 1 with their origin and topological locations. The mutations shown were selected from the dozens identified on the basis that if two mutations acted at the same location in the structure, only one was studied further.

**Table 1:** Mutations that suppress the effect of the capsid destabilising mutation VP3 91F in the Sabin type 3 strain.

Mutation	Location	Strains where identified
VP2 18 leucine-isoleucine	Beta sheet at pentamer interface	Isolates from vaccinees and in vitro passage at elevated temperature
VP2 215 leucine-methionine	Protomer interface	Isolates from vaccinees
VP2 241 aspartate-glutamate	VP2/VP3 interface, buried	In vitro passage at elevated temperature
VP3 19 histidine-tyrosine	Internal network, β-annulus beneath 5 fold axis	In vitro passage at elevated temperature
VP3 85 leucine-phenylalanine	Beta sheet at pentamer interface	In vitro passage at elevated temperature
VP3 91 phenylalanine -serine	Protomer interface	Isolates from vaccinees and in vitro passage at elevated temperature
VP1 54 alanine-valine	Internal network at 3 fold axis	Isolates from vaccinees and in vitro passage at elevated temperature
VP1 132 phenylalanine -leucine	Capsid pocket	In vitro passage at elevated temperature

#### (ii) Type 3 Leon strain

Mutations identified as candidate capsid stabilising changes were introduced into Leon, the virulent precursor of the Sabin vaccine strain of type 3 in different combinations. It might be expected that if the capsid were stabilised beyond what is optimal for assembly or uncoating, growth would be slowed down, and in fact viruses possessing three or more mutations grew significantly more slowly than the parental Leon when recovered and grown in HEp2c cells at 35°, a temperature fully permissive for the wild type strain. On passage at 37°C, faster growing variants emerged which had lost one or more of the stabilising capsid mutations or introduced additional mutations including one in VP4 at residue 67 where a threonine replaced an alanine and another in VP1 at residue 105 where a threonine replaced a methionine, in both cases as found in the wild type 3 virus Saukett (Table 2). As these changes rescued a virus with an over-stable structure it was concluded that the amino acids introduced had a destabilising effect. Thus structures with the parental version of these residues (VP4 67A and VP1 105M) were thought likely to be more stable than those that did not.

**Table 2:** Mutations that destabilise capsids in super-optimally stable Leon mutants and are also present in wild type Saukett.

Mutation	Location
VP4 67 alanine* -threonine	Internal network near three fold axis
VP1 105 methionine* -threonine	North wall of canyon

*amino acid present in stable capsid

#### (iii) Mutations in the type 3 strain Saukett -SC8

The strain that is used for production of inactivated serotype 3 polio vaccine, hence the appropriate target for VLP design, is the virulent, wild-type virus Saukett, whose capsid proteins differ from those of Leon at 14 amino acid positions, some in antigenic sites. Saukett already has the thermostable version of two of the mutations identified in the Sabin type 3 strain (VP3 91 serine and VP1 54 valine) and also possesses the destabilising residue of the two mutations identified by passage of the Leon constructs. Constructs were therefore made in which the remaining 6 amino acids in Table 1 and the 2 mutations in Table 2 were exchanged for the stabilising forms, so giving Saukett-SC8, with eight amino acid differences from the IPV Saukett sequence as shown in Table 3 which also describes the nature of their locations within the virus structure.

**Table 3:** Mutations included in capsid stabilised mutants for further study: Mahoney-SC7, MEF-SC5a and Saukett-SC8.

Virus	Mutation	Location
**Mahoney-SC7 (type 1)**	R4018G	Internal network near three fold axis
	T2025A	Pentamer interface
	D2057E	Pentamer interface
	L3119M	VP2/VP3 interface
	Q3178L	Protomer interface
	V1196L	Pocket
	H1248P	Protomer interface
**MEF-SC5a (type 2)**	L3085F	Beta sheet at pentamer interface
	Q3178L	Protomer interface
	T1041I	Pentamer interface
	F1134L*	Pocket
	Y1159F	Pocket
**Saukett-SC8 (type 3)**	T4067A	Internal network near three fold axis
	L2018I	Beta sheet at pentamer interface
	L2215M	Protomer interface
	D2241E	VP2/VP3 interface, buried
	H3019Y	Internal network, tube below 5 fold axis
	L3085F	Beta sheet at pentamer interface
	T1105M	North wall of canyon
	F1132L*	Pocket

* F1134L in type 2 is equivalent to F1132L in type 3

#### (iv) Mutations in type 1 Mahoney-SC7 and type 2 MEF-SC5a strains

The similarities in the structures of type 1, 2 and 3 poliovirus suggested that the stabilising mutations identified for type 3 might also have a stabilising effect on types 1 and 2. However the type 1 Mahoney strain used in most current IPV production already possesses four of the mutations incorporated into Saukett-SC8. The remaining four were introduced in addition but the construct produced no detectable capsid proteins. A second identification strategy was therefore followed, which involved constructing a mutant of Mahoney possessing a phenylalanine at residue 91 of VP3, as in the Sabin 3 strain; this virus was recovered and shown to be temperature sensitive in its growth in vitro. HEp2c cells were transfected with RNA transcripts from this construct, incubated at the non-permissive temperature of 39°C until at least 80% cytopathic effect (CPE) was seen, and the resulting progeny examined by deep sequencing to identify subpopulations of non-temperature sensitive mutants. The transfection was independently repeated six times and each population subject to deep sequencing twice to eliminate amplification artefacts. Some of the mutations identified in this way were inserted into Mahoney to give Mahoney-SC7 and are shown in Table 3.

The same strategy was followed for the type 2 strain MEF-1 and the mutations identified and inserted into MEF-SC5a are given in Table 3. Reversion of VP3-91 (F-S) occurred in a significant proportion of both populations.

**Fig 1:**
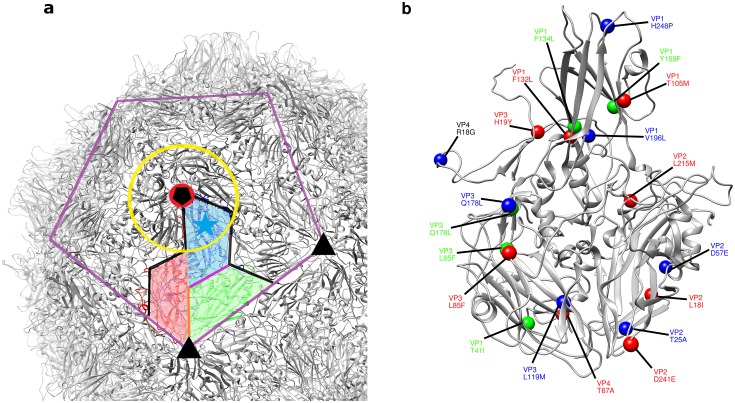
Location of stabilising mutations in three poliovirus stereotypes. (a) A cartoon depiction of the type 1 (Mahoney strain) poliovirus capsid in grey focussing on the area surrounding an individual protomeric subunit (protein chains coloured VP1 blue, VP2 green and VP3 red). The capsid features described in Table 3 are identified on the capsid as follows—yellow ring: canyon, cyan star: VP1 pocket, purple line: pentamer interface, black pentagon: five-fold axis, black triangle: three-fold axis, red circle VP3 beta-annulus, orange line: VP2/VP3 interface, black line: protomer interface and magenta line: VP1/VP2 interface. (b) an enlarged protomeric subunit in grey with the mutations for all three stereotypes shown color-coded as: blue: Mahoney-SC7 (type 1), green: MEF-SC5a (type 2) and red: Saukett-SC8 (type 3).

The positions of all the altered residues within a protomeric subunit and the locations of the relevant capsid features are shown in Fig 1. Importantly, none of the candidate stabilising mutations selected in any of the three serotypes occurred at residues previously identified as contributing to antigenic sites. They were therefore thought unlikely to alter the antigenic structures of the particles in which they were present.

### Infection by candidate thermostable mutants

**Fig 2:**
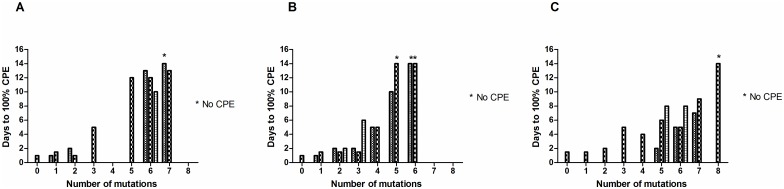
Stabilisation of virus particles reduces infectivity. Different numbers and combinations of the stabilising mutations described in Tables 1 & 3, as well as others identified in similar ways, were introduced into capsid-coding sequences of infectious clones. HEp2c cells were transfected with infectious RNA transcripts and incubated at 37°C until 100% cytopathic effect (CPE) was observed, or frozen after 7 days if no CPE was apparent; clarified supernatants of these cell cultures were blind passaged into fresh HEp2c cells and cells incubated at 37°C until 100% CPE was observed or for a further 7 days. (A) type 1 Mahoney capsid mutants, (B) type 2 MEF-1 capsid mutants, (C) type 3 Leon capsid mutants. * no CPE observed after blind passage.

Genomes were constructed that included varying numbers of the candidate mutations identified and RNA was transfected into HEp2C cells which are permissive for polio growth. The results obtained are shown in Fig 2 where it can be seen that the time to full lysis of the cell culture at 37°C increased with the number of mutations inserted until no CPE could be detected at all in some cases, even after blind-passage (SC8 containing 8 mutations in the wild type Leon for type 3, SC5b, SC6a & SC6b containing 5 or 6 mutations in the wild type MEF-1 for type 2 and SC7 containing 7 mutations in the wild type Mahoney for type 1). At 33°C incubation times were even longer. Transfection of the type 3 construct Saukett-SC8, like Leon SC8, did not result in any detectable CPE. The properties of Saukett-SC8 (type3), MEF-SC5a (type 2) and Mahoney-SC7 (type1) were examined further.

### Thermostability of native-antigenic particles produced by transfection

Poliovirus particles express two distinct antigens. D antigen is associated mainly with infectious virus and C antigen with non-infectious particles in a different conformation, for example resulting from heating; wild-type empty capsids, outside the cell, are particularly prone to convert to C antigen specificity. IPV potency is expressed in D antigen units as D antigen is thought to be the inducer of a protective immune response. An ELISA developed for quantitating the D antigen content of commercial vaccines [8, 12, 13] was adapted for measuring the D antigen or C antigen content of virus particle preparations, based on the use of D antigen specific monoclonal antibodies against types 1, 2 and 3 [14], and C antigen specific antibodies for types 1 and 3. No C specific antibody was available for type 2.

**Fig 3:**
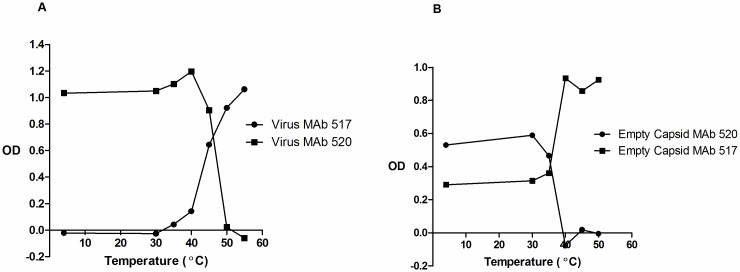
Thermostability of type 3 Leon particles. Reactivity of (A) virus and (B) empty capsid aliquots with MAb 520 and MAb 517 in ELISA after incubation at different temperatures for 10’. MAb 520 is specific for D Antigen and MAb 517 is specific for C Antigen.

L cells, which lack the receptor for poliovirus, were electroporated with full length RNA transcripts from constructs encoding the wild type or capsid stabilised mutants to give a single cycle of infection. Overnight incubation produced predominantly C antigenic empty particles for wild type constructs so 6 hour incubations were used in these cases; production of predominantly D antigenic empty particles for mutant constructs after overnight incubation was an early indication that the introduced mutations had a significant stabilising effect. The particles produced were purified on sucrose gradients and the fractions were screened by ELISA to identify the relevant peaks. Type 1 and type 3 peak fractions were also screened by immunoblotting with a VP2 specific antibody. Virion fractions contained VP2 and empty capsid fractions contained VP0, as expected. The fractions corresponding to the virions and empty capsids were exposed to a range of temperatures for ten minutes and assayed for D antigen content, and C antigen where possible. The results for the unmodified type 3 strain Leon are shown in Fig 3A for the infectious virus and in Fig 3B for the empty capsids where there was a high starting background of C antigen as expected. The loss of D antigen was mirrored by an increase in C antigen content; for the infectious particles the temperature at which 50% of D antigen was lost was 42°C while it was 33°C for the empty capsids.

The results for empty capsid preparations of all wild type and selected stabilised capsid mutants are summarised in Table 4. The modified empty capsids converted from D to C antigen specificity at 54°C, 56°C and 55°C for the stabilised forms of types 1, 2 and 3 respectively compared to 36°C, 42°C and 33°C for the corresponding wild type strains. The temperatures at which commercial IPV converted from D to C antigen specificity ranged from 49°C to 52°C for the three serotypes., Therefore, by this assay, all of the empty capsids from the mutant strains selected for improved thermostability were more stable than the currently marketed IPV produced by formalin treatment of live virus.

**Table 4:** Thermostability of empty capsid (VLP) preparations.

Particle		Temperature*
Native empty capsid (WT1)		36°C
Native empty capsid (MahSC7)		54°C
Native empty capsid (WT2)		42°C
Native empty capsid (MEFSC5a)		56°C
Native empty capsid (WT3)		33°C
Native empty capsid (SktSC8)		55°C
IPV (formaldehyde treated)	Type 1	49°C
	Type 2	52°C
	Type 3	52°C

*–at which native antigenicity is reduced by 50% after a 10’ incubation

Assays using different D antigen specific MAbs directed against other antigenic sites gave indistinguishable results and all the antibodies tested reacted equally with wild type and stabilised particles in ELISA.

**Fig 4:**
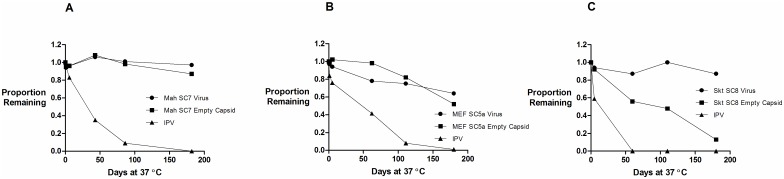
Long-term stability of virus and empty capsid preparations compared to the IPV reference. Proportion of D antigen reactivity remaining after incubation at 37°C relative to incubation at 4°C. Aliquots of IPV, virus and empty capsid samples were incubated at 37°C and samples were removed at intervals and analysed by D Antigen ELISA; reactivity is expressed relative to samples incubated at 4°C for the same period. (A) Type 1, (B) Type 2, (C) Type 3.

The study involved exposure at elevated temperature for ten minutes. A study that would better imitate real use involved exposing the materials to 37° for prolonged periods; the results are shown in Fig 4 for the D antigen loss, in comparison with IPV, of both the empty capsid peak and the virus peak relative to samples incubated at 4°C for the same period. The 4°C samples showed no significant loss of reactivity throughout the incubation period and were used as controls for inter-assay variability. For type 3 (Fig 4C) IPV had lost all activity by the second time point at 62 days; it is possible that total loss occurred much earlier. The type 3 Saukett-SC8 virus retained full activity for 180 days and the empty capsids of Saukett-SC8 (SktSC8 VLP) retained 56% of the activity at 62 days and 13% at 180 days. The type 2 component of IPV (Fig 4B) lost D antigen content less quickly than observed for type 3 but only retained 41% on day 62 and 8% by day 111. The virus particle of the mutant type 2 virus MEF-SC5a retained 75% of starting D antigen content on day 111, and retained 64% at day 180. The empty capsids of MEF-SC5a retained 82% of starting D antigen content by day 110 and 52% of the activity at 180 days. The type 1 component of IPV (Fig 4A) retained 35% of D antigen content by day 43 and only 4% by day 86; reactivity was abolished by day 182. Neither the virus particle nor the empty capsids of the mutant type 1 virus Mah-SC7 lost any D antigen content by day 86; the empty capsids of MEF-SC7 retained 87% of D antigen content on day 182. The stabilised empty capsids were therefore strikingly more stable than IPV at 37°. If this were also true of a commercial product it could survive well outside the cold chain.

### Immunogenicity in rats

It is known that the type 2 component of Sabin IPV is less immunogenic than the wild type 2 strain in classical IPV [6], an observation that has not been explained in a satisfactory way. It was therefore necessary to investigate the immunogenicity of empty capsids (or VLPs) of the stabilised strains to compare them to classical IPV.

The immunogenicity assay of commercial IPV involves measuring the antigen content, usually by ELISA as above, and then immunising rats with a range of dilutions based on the human dose [13]. The proportion of animals seroconverting at a specific cut off in neutralisation assays is compared to that seen with a reference preparation tested at the same time and a relative potency can be calculated. In this test the type 2 strain used in most current production induces a greater serological response than the other two serotypes and the cut off is therefore far higher. Results for type 1, 2 and 3 are shown in Table 5.

**Table 5:** Immunogenic potency of strains in rats.

	Type 1: Proportion of animals responding at a dilution endpoint titre of >4 to:			
	32 D Antigen Units	16 D Antigen Units	8 D Antigen Units	4 D Antigen Units
Type 1 IPV	9/10	5/10	2/10	1/10
MahSC7 VLP	10/10	10/10	10/10	10/10
	Type 2: Proportion of animals responding at a dilution endpoint titre of >512 to:			
	8 D Antigen Units	4 D Antigen Units	2 D Antigen Units	1 D Antigen Unit
Type 2 IPV	8/10	8/10	2/10	2/10
MEFSC5a VLP	10/10	9/10	8/10	6/10
	Type 3: Proportion of animals responding at a dilution endpoint titre of >4 to:			
Sample	28 D Antigen Units	14 D Antigen Units	7 D Antigen Units	3.5 D Antigen Units
Type 3 IPV	9/10	7/10	2/10	3/10
SktSC8 VLP	10/10	10/10	10/10	10/10

The thermostable VLPs MahSC7 (type 1) and SktSC8 (type 3) caused seroconversion in all animals at all doses given, whereas the responses in animals given the same doses of classical IPV spanned the 50% response dose. For type 2 the responses in animals given IPV spanned the 50% end point and the potency of the SC5a VLPs was at least four fold higher. The stabilised VLPs are therefore at least four times more immunogenic than the equivalent IPV component.

### Protection from challenge

**Fig 5:**
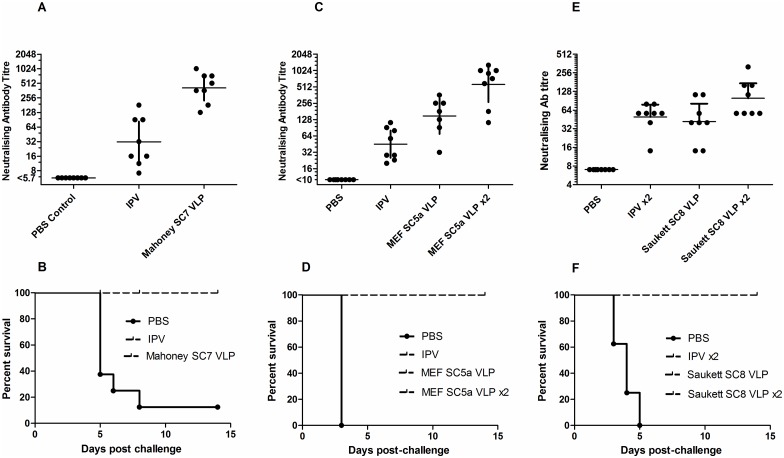
Seroconversion and protection against challenge induced by VLPs. Transgenic mice expressing the human poliovirus receptor were immunised intraperitoneally once or twice (x2) with PBS or 0.5 human dose equivalents of IPV or VLPs (A, B—type 1; C, D—type 2; E, F—type 3) then challenged intramuscularly with 25 PD_50_ of homologous wild type virus. Graphs A, C & E show neutralising antibody titres against homologous serotype viruses in blood samples taken the day prior to challenge. Graphs B, D & F show survival rates following challenge with (B) type 1 Mahoney, (D) type 2 MEF-1 and (F) type 3 Saukett. Bars (A, C & E) indicate 95% CI of the geometric mean titre.

Transgenic mice carrying the human receptor for poliovirus are susceptible to infection and paralysis. Groups of animals were immunised with one or two doses of D antigen corresponding to half a human dose for the stabilised VLPs, or IPV. They were then challenged with 25 PD_50_ of the Mahoney type 1 strain, the MEF-1 type 2 strain or the Saukett type 3 strain. The results are shown in Fig 5A–5F which also show the pre-challenge neutralising antibody titres. In all cases the stabilised capsids given as either one or two doses were more immunogenic than IPV and protected all animals from challenge with the corresponding virulent virus.

## Discussion

We reported the production of empty capsids by expression of poliovirus proteins in recombinant baculovirus-infected insect cells that did not involve poliovirus growth more than two decades ago but the particles were too unstable to be useful as a vaccine candidate [15]. Immunogenic, but unstable, VLPs were also produced by recombinant expression in yeast [16]. A strategy to address this instability is described here where candidate stabilising mutations were identified then multiple changes introduced into capsid proteins in combination. This approach took advantage of a mutation known to be present in the type 3 Sabin vaccine strain of poliovirus that destabilises capsid assembly intermediates (including empty capsids) without affecting virion stability [10]. Thus revertants from vaccinees and other sources were thought likely to possess mutations that restore assembly and increase empty capsid stability. Stabilising mutations in the type 1 and 2 strains were identified by inserting the destabilising Sabin type 3 mutation, selecting in transfected cells at non permissive temperature and screening for revertant genomes by deep sequencing. The approach could also be used to guide selection for other, non-polio, picornaviruses. In some cases stabilising mutations found for type 3 were already present in the type 1 and type 2 viruses. The work described here was performed as part of a consortium whose members are given under Acknowledgments; the consortium is also following other approaches to the identification of candidate capsid stabilising mutations including prediction of stabilising interactions from the atomic structure and selecting for thermostable virus mutants by limited exposure to high temperatures. The consortium is also developing several recombinant expression systems for virus-free synthesis of stable, immunogenic VLPs.

The structural locations of some of the mutations responsible for stabilising empty capsids here have been described [9]; locations of the other mutations (Tables 1–3, Fig 1) were analysed in similar ways by inspection of atomic resolution structures of Mahoney [17], Lansing [18] and Sabin 3 [9]. In most cases these locations provided insight into their mechanisms of action. Thus, some mutations appear to act by stabilising interfaces between particle subunits; others may stabilise contacts within subunits including those with the “pocket factor”, thought to be a short chain fatty acid that is present in all polioviruses that acts to stabilise particles against conformational transitions [9, 19]; yet others plausibly strengthen interactions between subunits in the internal network that hold subunits together, such as the tube-like beta-annulus structure beneath the 5-fold axis [17, 18, 9]. Undoubtedly, mutations other than those described here exist that can similarly stabilise polio empty capsid particles and evidence to support this will be presented elsewhere. The overall strategy may be useful in the development of stable VLP vaccines for other picornaviruses.

Licensing and regulation of a new vaccine would normally require a clinical trial that demonstrates efficacy against the target disease. This is clearly impractical for a new polio vaccine where the number of cases in the world is approaching zero. There are precedents for other approaches; Sabin based IPV is licensed in China and Japan on the results of immunogenicity studies and guidance is provided in WHO guidelines on the types of clinical and preclinical trials that might be done [20]. Meningitis C vaccine was licensed and implemented in the UK on the basis of immunogenicity data from clinical trials, and Human Papilloma vaccines were licensed on their ability to prevent non-cancerous lesions not cervical cancer [21]. The decision on whether to license a vaccine lies with the National Regulatory Authorities (NRAs) rather than WHO, but these examples prove that it is possible to gain approval without direct evidence of clinical efficacy from studies showing protection from natural challenge.

The final preparations studied were extremely stable compared to IPV and could conceivably give rise to a vaccine that would not require a cold chain; moreover they were more immunogenic than IPV made from the equivalent strains in the animal model used for testing IPV potency and in challenge studies in transgenic mice. It is possible that this is partly because the particles, unlike IPV, were not treated with formalin. The viruses from which they were derived had lost infectivity presumably because they were unable to uncoat by virtue of their hyperstable capsids. A suitable expression platform would be required to make this a viable vaccine production system and is the focus of one strand of current work of the Consortium, but the properties of the particles are very promising.

## Materials and Methods

### Construction and recovery of variants

Derivation of the pT7/Leon cDNA clone has have been described previously [22]. A ribozyme sequence was inserted between the T7 promoter and the polio cDNA so that RNA transcripts began with the authentic 5’ end. In order to replace the P1 coding region a SacII site was introduced without coding change at nucleotides 3408–13 as described [7]. Capsid regions from Mahoney, MEF-1 and Saukett were then introduced precisely using standard PCR methods. Mutations were introduced into the capsid protein coding regions of these clones using synthetic DNA and suitable restriction enzyme cleavage sites.

Viruses were recovered by transfection of HEp2C monolayers in 25cm^2^ flasks with 2μg T7 transcripts [23] followed by incubation at 33°, 35° or 37°C until complete cytopathic effect (CPE) was apparent; transfected cells showing no signs of CPE at 7 days were frozen and cell lysates were blind-passaged on fresh HEp2c cells for a further 7 days. HEp2c cells were from stocks maintained at NIBSC since the 1970s.

### Particle purification

Particle preparations were made by high-efficiency electroporation of full-length RNA transcripts into mouse L cells. L cells were from stocks maintained at NIBSC. Linearised clones (1μg) were transcribed using a T7 Megascript kit (Life Technologies); cells from a 90% confluent 75cm^2^ flask were removed by trypsinisation, washed and resuspended in HeBS (20mM HEPES, 137mM NaCl, 5mM KCl, 0.7mM Na_2_HPO_4_, 6mM glucose, pH 7.05), electroporated with RNA transcripts (250V, 250μF, 360Ω) then returned to the flask and incubated in DMEM at 37°C overnight or for 6h. Six flasks were used for each construct. Cell sheets were then frozen at -20° and thawed, and cell debris removed by centrifugation. Igepal was added to supernatants to a final concentration of 0.1% and viral particles were concentrated by centrifugation at 4°C through a 10ml 30% sucrose cushion made up in 50mM NaCl and 10mM Tris HCl pH7.2. Pellets were resuspended in 1ml 6-salt PBS per six flasks, layered on 10ml 15%- 30% sucrose gradients in lysis buffer (6-salt PBS containing 0.5% sodium deoxcholate, 20mM EDTA and 1% Igepal) and centrifuged at 4°C for12h at 18,000rpm in a Beckman SW41 rotor before harvesting into 20 0.5 ml fractions. Fractions were screened by C or D antigen specific ELISA or by immunoblotting (only for type 1 and 3) to identify virus and empty capsid peaks. Primary antibodies for immunoblots were rabbit anti-VP2 peptide sera R271 for type 1 and R268 for type 3 [24].

### D and C antigen ELISA

A non-competitive sandwich ELISA assay was used to measure the D antigen content of poliovirus [12]. Briefly, two-fold dilutions of antigen were captured with a serotype-specific polyclonal antibody, then detected using serotype-specific, D antigen- or C antigen-specific monoclonal antibodies followed by anti-mouse peroxidase conjugate. The D antigen content of each test sample was evaluated against a reference of assigned D antigen content [23] by parallel line analysis (Combistats). For D antigen specific ELISA the monoclonal antibodies used were 234 for type1, 1050 for type 2 and 520 for type 3, and for C antigen specific ELISA 1588 for type 1 and 517 for type 3. No C specific type 2 antibody was available.

### Thermostability

The temperature at which a conformational change from D to C antigenicity occurred was determined by heating at a range of temperatures from 30–60°C followed by D and C antigen ELISA (the latter where possible). Samples were diluted in 6-salt PBS to twice the concentration required to obtain an OD of 1.0 in D antigen ELISA, duplicate samples heated for 10min at each temperature then diluted 1:1 with 4% dried milk in 6-salt PBS and cooled on ice. D and C antigen content was measured by ELISA.

Long-term stability was analysed by incubating, at 37°C, multiple aliquots of IPV, virus and empty capsid samples diluted in 6-salt PBS to twice the concentration required to obtain an OD of 1.0 in D antigen ELISA. Samples were removed at intervals and analysed by D Antigen ELISA.

### Immunogenicity

Immunogenicity was assessed using Pharmacopeial methods established at NIBSC for the release of IPV lots. D antigen content was measured by ELISA and immunogenicity was assessed in Wistar rats [12]. Readout was based on the proportion of animals having a neutralisation titre above a predetermined cut off as given in the tables.

### Immunisation- challenge

TgPVR mice of both sexes (8 per test group) received one or two intraperitoneal injections of PBS (controls) or the equivalent of 0.5 human doses of purified VLPs or the IPV European reference BRP [25]. VLP preparations were shown to be non-infectious by inoculation of HEp2c monolayers and blind-passage after 7 days. The second dose, where given, was on day 14. On day 35 blood samples were taken and mice were challenged intramuscularly with the equivalent of 25 times the PD_50_ of the relevant serotype of wild type poliovirus (Mahoney, MEF-1 or Saukett) then monitored for any signs of paralysis for 14 days [8].

### Deep sequencing of virus harvests

RNA was extracted using Roche High Pure viral RNA kits. Water only controls were extracted, amplified and sequenced in parallel with each set of samples. Capsid coding regions were amplified in duplicate by one-step RT-PCR using a SuperScript III HiFi kit and primers P1F (5’-GCGAGTTGGATTGGCCATCCAGTG -3’) and P1R (5’-TGGAAGGTGGGTCCCACAAACGAC-3’). Products were purified using AMPure XP magnetic beads (Beckman Coulter), quantified using Qubit High Sensitivity dsDNA assay (Life Technologies), analysed on an Agilent High Sensitivity DNA chip (Agilent) and diluted to 0.2 ng/μl in molecular grade 10mM Tris–EDTA, pH8.0.

Sequencing libraries were prepared using Nextera XT reagents (Illumina) and the manufacturer's protocol, and sequenced on a MiSeq using a 2 × 251 paired-end v2 Flow Cell (Illumina). Quality trimming and assembly were carried out as in Mee at al. [26]. Reads were then mapped to parental reference sequences using Geneious R7 (Biomatters) software and SNPs present at ≥ 0.5% identified. Only those SNPs present in both replica amplicons were retained.

### Ethics statement

All animal experiments were performed under licenses granted by the UK Home Office under the Animal (Scientific Procedures) Act 1986 revised 2013 and reviewed by the internal NIBSC Animal Welfare and Ethics Review Board. The TgPVR mouse and rat immunogenicity experiments were performed under Home Office licences PPL 80/2478 and PPL 80/2050 which were reviewed and approved by the NIBSC Animal Welfare and Ethics Review Board before submission.
